# Rewarding properties of L-Dopa in experimental parkinsonism are mediated by sensitized dopamine D1 receptors in the dorsal striatum

**DOI:** 10.1038/s41380-024-02721-3

**Published:** 2024-09-03

**Authors:** Carina Plewnia, Débora Masini, Gilberto Fisone

**Affiliations:** 1 Department of Neuroscience, Karolinska Institutet, 17177 Stockholm, Sweden; 2 Present Address: Department of Biochemistry and Biophysics, Stockholm University, 10691 Stockholm, Sweden

**Keywords:** Neuroscience, Addiction

## Abstract

Treatment of Parkinson’s disease (PD) is based on the use of dopaminergic drugs, such as L-Dopa and dopamine receptor agonists. These substances counteract motor symptoms, but their administration is accompanied by motor and non-motor complications. Among these latter conditions a neurobehavioral disorder similar to drug abuse, known as dopamine dysregulation syndrome (DDS), is attracting increasing interest because of its profound negative impact on the patients’ quality of life. Here we replicate DDS in a PD mouse model based on a bilateral injection of 6-hydroxydopamine (6-OHDA) into the dorsal striatum. Administration of L-Dopa induced locomotor sensitization and conditioned place preference in 6-OHDA lesion, but not in control mice, indicative of the acquisition of addictive-like properties following nigrostriatal dopamine depletion. These behavioral effects were accompanied by abnormal dopamine D1 receptor (D1R) signaling in the medium spiny neurons of the dorsal striatum, leading to hyperactivation of multiple signaling cascades and increased expression of ΔFosB, a stable transcription factor involved in addictive behavior. Systemic administration of the D1R antagonist, SCH23390, abolished these effects and the development of place preference, thereby counteracting the psychostimulant-like effect of L-Dopa. The rewarding properties of L-Dopa were also prevented by chemogenetic inactivation of D1R-expressing neurons in the dorsal striatum. Our results indicate the association between abnormal D1R-mediated transmission and DDS in PD and identify potential approaches for the treatment of this disorder.

## Introduction

Parkinson’s disease (PD) is classically defined by the progressive loss of dopamine neurons located in the substantia nigra pars compacta (SNc) and projecting to the dorsal striatum, and by the emergence of rigidity, tremor, and bradykinesia [1, 2]. These motor symptoms are commonly treated with dopamine replacement therapy (DRT). However, the prolonged use of L-Dopa and dopamine receptor agonists leads to the development of motor (i.e., dyskinesia) and non-motor complications [3]. These latter conditions include a neurobehavioral disorder similar to drug abuse, referred to as dopamine dysregulation syndrome (DDS). DDS is more frequently observed in patients treated with short-acting drugs, such as L-Dopa or apomorphine, and is characterized by a pathological overconsumption of dopaminergic medications, far beyond that necessary to correct motor disabilities. DDS patients feel under-medicated, ignore advised dose schedules and self-medicate to a state where they only feel “on” when notably dyskinetic [4–7]. This psychiatric condition represents a major problem for the patients and their relatives and seriously limits the use of drugs commonly employed to manage PD. Whereas the study of the motor complications associated with administration of L-Dopa has been the subject of intense research [8], less is known about the mechanisms implicated in the non-motor side effects caused by this drug.

In PD, administration of L-Dopa regulates the activity of two major populations of striatal projection neurons, referred to as medium spiny neurons (MSN), expressing dopamine D1 or D2 receptors (D1R and D2R). A large body of evidence indicates that the depletion of dopamine occurring in PD leads to enhanced sensitivity of D1R expressed in MSN [8–10]. In experimental PD, this phenomenon leads to the hyperactivation of multiple signaling pathways, including the cAMP, extracellular signal-regulated kinase (ERK) and mammalian target of rapamycin (mTOR) cascades, ultimately resulting in long-term modifications of gene expression and protein synthesis. In the striata of rodent and non-human primate models of PD, the transcription factor ΔFosB, a stable splice variant of the immediate early gene FosB, is up-regulated following chronic L-Dopa treatment. These signaling abnormalities have been linked to the emergence of motor complications, such as levodopa-induced dyskinesia [8, 9, 11–13]. Importantly, enhanced expression of ΔFosB has been implicated in the long-term effects produced by substances of abuse and natural rewards [14, 15] but its involvement in the acquisition of motor programs at the basis of compulsive behavior in DDS remains to be assessed.

L-Dopa gained rewarding properties in a rat model of PD with a partial loss of dopamine neurons in the SNc induced by viral-mediated expression of α-synuclein [16]. In line with this finding, apomorphine showed a higher propensity to develop psychostimulant-like properties in rats with a 6-hydroxydopamine (6-OHDA) lesion of nigrostriatal dopamine neurons [17]. These studies indicate the possibility of using rodent preclinical models to identify pathological changes responsible for DDS.

In this study, we reproduced features of DDS in a mouse model of PD based on bilateral injection of the neurotoxin 6-OHDA in the dorso-lateral striatum, which leads to a partial degeneration of midbrain dopamine neurons [18]. The rewarding effect of L-Dopa was examined in the conditioned place preference (CPP) and locomotor sensitization paradigms, in parallel to biochemical analyses of different components of the D1R signaling cascade. Pharmacological and chemogenetic inhibition of D1R was tested for its ability to counteract DDS-like behavior and associated biochemical changes produced by L-Dopa.

## Material and methods

### Animals

Studies were performed in adult (2–4 months old) mice of both sexes. We used C57BL/6J mice (20–25 g; Charles River, Sulzfeld, Germany) and mice expressing enhanced green fluorescent protein (EGFP) under the control of the promoter for D2R (D2-EGFP mice) [19]. Chemogenetic experiments were performed in heterozygous Drd1a-Cre (D1-Cre) transgenic mice (EY262 line, GENSAT project). All mice were acclimatized for one week before surgery. Mice were group-housed under a 12:12 h light-dark cycle with access to food and water ad libitum. Experiments were performed in accordance with the guidelines of Research Ethics Committee of Karolinska Institutet, Swedish Animal Welfare Agency, and European Communities Council Directive 86/609/EEC.

### Drugs

6-Hydroxydopamine hydrochloride (6-OHDA, Sigma-Aldrich, Stockholm, Sweden) was dissolved in 0.02% ascorbic acid and 0.9% sterile saline at a free base concentration of 4 μg/μl and injected in the dorso-lateral striatum. 3,4-dihydroxy-l-phenylalanine (L-Dopa, 10 mg/kg in sterile saline; Sigma-Aldrich, Stockholm, Sweden) was administered intraperitoneally (i.p.) together with the DOPA decarboxylase inhibitor benserazide hydrochloride (7.5 mg/kg in saline; Sigma-Aldrich, Stockholm, Sweden) at a volume of 10 ml/kg bodyweight. Mice were treated with L-Dopa (+Benserazide) alone or in combination with selective inhibitors: SCH23390 (SCH, 0.25 mg/kg in 0.9% sterile Saline; Tocris‐Biotechne Ltd., Abingdon, UK) was administered i.p. 10 min before L-Dopa at 10 ml/kg bodyweight. Rapamycin (Rapa, 5 mg/kg in 5% DMSO, 5% Tween20, 15% PEG in dH2O; LC Laboratories, Woburn, USA) and PD0325901 (PD03, 5 mg/kg in 10% DMSO, 5% Tween20, and 15% PEG in dH20; Selleckchem, Planegg, Germany) were injected i.p. at 2 ml/kg and 45 min before L-Dopa injection. For chemogenetic experiments, Clozapine N-oxide (CNO, 1 mg/kg in 5% DMSO in 0.9% sterile saline; Tocris‐Biotechne Ltd., Abingdon, UK) was administered at 10 ml/kg bodyweight 20 min before L-Dopa. Control mice were injected with vehicle, accordingly.

### Stereotaxic surgery

All surgical procedures were performed in 2.5–3-month-old mice. Animals received a bilateral partial lesion with the neurotoxin 6-OHDA using a well-established protocol [18]. Briefly, mice were anesthetized with isoflurane and positioned in a stereotaxic frame (Stoelting, Wood Dale, Il, USA) equipped with a heating pad to maintain normothermia. Animals were injected subcutaneously with an analgesic (0.1 mg/kg Temgesic; Apoteket, Stockholm, Sweden) and anesthetic cream (EMLA, 2.5% lidocaine, 2.5% prilocaine; Apoteket, Stockholm, Sweden) was applied locally. Mice received ophthalmic ointment to prevent corneal dryness. 6-OHDA hydrochloride was freshly dissolved and 1.25 µl was injected into each dorsal striatum at a rate of 0.2 µl/min according to the following coordinates (mm from bregma): anteroposterior +0.6; medio-lateral ±2.2 and dorsoventral −3.2. The injector was left in place for an additional 5 min, allowing the solution to diffuse. Control (sham) mice received bilateral injections of 1.25 μl vehicle. Mice were allowed to recover for 3 weeks before experiments. We followed an enhanced pre- and post-operative care protocol to minimize mortality to <5% [18]. For chemogenetic experiments D1-Cre (+/-) or wildtype (-/-) mice were injected with 6-OHDA bilaterally (CPP experiment), or unilaterally (rotation test), following the same protocol as described above. After 3 weeks recovery, mice received bilateral (CPP experiment) or unilateral (rotation test) infusions of a Cre-inducible-adeno-associated virus carrying the gene for the inhibitory Designer Receptors Exclusively Activated by Designer Drugs (Gi-DREADD) and were allowed to recover for three more weeks before behavioral experiments. The Gi-DREADD (pAAV5-hSyn-DIO-hM4D(Gi)-mCherry; titer ≥7 × 1012 vg/mL, Addgene plasmid #44362) was injected (0.5 µl per hemisphere) at a rate of 0.1 µl/min according to the following coordinates (mm from bregma): anteroposterior +0.6; medio-lateral ±2.2 and dorsoventral −3.2.

### Conditioned place preference

CPP is a form of Pavlovian conditioning used to assess the rewarding and motivational properties of addictive drugs [20]. The effect of L-Dopa was tested in a CPP paradigm performed over 8 consecutive days. The apparatus consisted of a white acrylic two-compartment chamber (20 × 20 × 50 cm each compartment). The two compartments were separated by detachable doors and differed in tactile cues on the floor (grooved vs. smooth). CPP procedures consisted of 3 phases: pre-conditioning, conditioning, and post-conditioning. During pre-conditioning, mice were given free access to both compartments for 15 min. Spontaneous preference was evaluated by measuring the average time each animal spent in either of the two compartments and animals spending >85% of time in one compartment were excluded from analysis due to a strong preference bias. During the conditioning phase, mice were injected with L-Dopa alone or in combination with SCH23390, rapamycin, PD0325901 or CNO (administered 10, 45, 45 or 20 min before L-Dopa, respectively) and immediately placed for 40 min in the “least-preferred” compartment (biased research design of CPP [21]). In the next session, mice received a vehicle injection and were placed in the opposite compartment. The procedure was repeated for a total of 12 times (6 days, 2 sessions per day). Accordingly, mice received 6 injections of drug paired to one specific compartment and the same number of vehicle injections paired to the opposite compartment. Control groups were injected with vehicle in both compartments during the entire conditioning phase. In the post-conditioning phase (24 h after the last conditioning session), mice were given free access to both compartments for 15 min in a drug-free state and the time spent in each of the two compartments was measured. In a separate experiment, CPP was examined 7 days after the last conditioning session. To test the effect of mTORC1 and ERK inhibitors on CPP memory recall [22], control mice and mice conditioned with L-Dopa alone received vehicle, rapamycin or PD0325901 45 min before the post-conditioning test. Reward-seeking behavior was assessed by comparing the time spent in the drug-paired compartment during pre-conditioning and post-conditioning stages. Preference score was calculated as post- minus pre-conditioning time spent in the drug-paired compartment. Behavior was video-recorded and analyzed by a video tracking software (Ethovision XT16, Noldus, The Netherlands).

### Locomotor activity

Locomotor activity was initially assessed during the CPP test by measuring the distance (m) moved during the 40 min daily conditioning trials. These results were further characterized by testing the mice in locomotor activity boxes (45 × 45 × 35 cm). In this experiment, locomotor activity was assessed in 15 min trials during 4 different phases: habituation, treatment, extinction and reinstatement. During habituation, mice were allowed to explore the arena in a drug-free state. In the treatment phase, locomotor activity was measured daily for 6 days following saline or L-Dopa injections. Extinction was measured 24 h and 7 days after the last treatment. Finally, mice received a priming injection of saline or L-Dopa and reinstatement of locomotor activity was assessed. Mice were video-recorded, and motor behavior was analyzed by a video tracking software (Ethovision XT16, Noldus, The Netherlands).

### Rotation behavior

Unilateral 6-OHDA lesion D1-Cre (+/-) or wildtype (-/-) mice expressing Gi-DREADD in the lesion hemisphere were placed in individual glass cylinders (12 cm diameter) and motor activity was video-recorded. Following a 20 min habituation, mice were injected with CNO and, 20 min later, with L-Dopa. The number of ipsilateral and contralateral rotations was manually counted for 90 min after L-Dopa injection by an observer blind to experimental groups.

### Western blot

Mice were sacrificed by decapitation, heads were immediately immersed in liquid nitrogen for 5 s, and the brains were rapidly removed. Tissue punches of dorsal and ventral striata from both hemispheres were dissected, sonicated in 1% SDS, and boiled for 10 min in sample buffer. The resulting homogenates were stored at −20 °C until further use. Aliquots of 2 × 5 µl of each homogenate were used for protein quantification with a BCA assay kit (Pierce, Rockford, IL, USA). Equal amounts of protein (25 µg/sample) were separated by SDS-PAGE and transferred overnight to nitrocellulose membranes (0.45 micron, Thermo Scientific, Germany). After the transfer, membranes were placed on a shaker, washed for 5 min in phosphate-buffered saline (PBS), and then blocked in Odyssey blocking buffer (Li-Cor Bioscience, Lincoln, NE, USA) for 1 h at room temperature. Next, membranes were incubated in primary antibodies: anti-tyrosine hydroxylase (TH; 1:2000, #AB152, Merck); anti-p44/42 MAPK ERK1/2 and anti-phospho-p44/42 MAPK ERK1/2 (1:2000, #9107 and #4377, Cell Signaling); anti-S6 ribosomal protein, anti-phospho-S6 ribosomal protein Ser 235/236 (1:1000, #2317 and #2215, Cell Signaling); anti-beta-actin (1:30000, #A5316, Merck); anti-FosB (1:1000, #sc-48, Santa Cruz Biotechnology) for two hours at room temperature and afterwards washed with PBS/0.1% Tween. Detection was based on fluorescent secondary antibody binding (IR Dye 800CW and 680RD; Li-Cor Bioscience, Lincoln, NE, USA), 1.5 h at room temperature. Quantification was performed with a Li-Cor Odyssey infrared fluorescent detection system, using the software Image Studio to quantify signal intensities. Data were calculated as % of control and phosphoproteins were normalized for the amount of the corresponding total protein detected in the sample or normalized for the amount of the corresponding beta-actin protein.

### Immunohistochemistry

Mice were anesthetized with pentobarbital (1:1 in 0.9% sterile saline) and perfused with 4% paraformaldehyde (PFA, Sigma-Aldrich, Darmstadt, Germany) in PBS (pH 7.4). Brains were dissected, post-fixed overnight in 4% PFA under continuous agitation at 4 °C, and cut into coronal sections (40 µm) using a vibratome (Leica VT 1000S, France). Tissue sections were stored at −20 °C in a solution containing 30% ethylene glycol, 30% glycerol and 0.1 M PBS until they were processed for immunofluorescence. Staining was performed on selected free-floating sections of the striatum and midbrain. Sections were permeabilized with PBS/0.1% Triton X-100 solution and blocked in PBS/0.3% Triton X-100 and 10% normal goat serum for 1.5 h. Sections were then incubated at 4 °C overnight with primary antibodies: anti-TH (1:1000, #AB152, Merck); anti-phospho-p44/42 MAPK ERK1/2 (1:1000, #4377, Cell Signaling); anti-phospho-S6 ribosomal protein Ser 235/236 (1:1000, #2215, Cell Signaling); anti-FosB (1:200, #sc-48, Santa Cruz Biotechnology); anti-GFP (1:1000, GFP-1020, AvesLabs); anti-RFP (1:1000; #600-401-379, Rockland) and subsequently with secondary antibody (1:500, Alexa Fluor 488, 594, 647; Jackson and ImmunoResearch) for one hour at room temperature. Finally, sections were mounted on poly-L-lysine prepared glass slides (Sigma diagnostic, USA) and covered with DABCO media (1,4-Diazabicyclo [2.2.2] octane powder in glycerol solution).

### Imaging and cellular counting

Representative images of the full striatum and midbrain were acquired at 10x (stitched tiling) and images for cell counting were acquired at 20x by confocal microscopy (LSM880, Zeiss, Germany) with the Zen software (Zeiss, Germany). Cell counts were performed in 3 sections per animal in the dorso-lateral striatum in proximity to the injection site or in 6 midbrain sections per animal, containing SNc and ventral tegmental area (VTA). Images were processed with ImageJ software (Scion Corporation, USA) and cell counting was performed by a researcher blind to treatment and groups.

### Statistical analyses

Sample size was calculated using G Power 3.1 [23] and was based on previously published studies. Data were analyzed using GraphPad Prism (Version 9, GraphPad Software, La Jolla, CA, USA) and tested for normality with the D’Agostino & Pearson test. Mice were randomly assigned to experimental groups and analysis was performed by a researcher blind to experimental groups. Data are presented as mean ± SEM unless stated otherwise. Statistical significance is presented as: *p < 0.05, **p < 0.01, ***p < 0.001. Figure legends contain p-values and number of animals and/or replicates. For 2-group comparisons, unpaired one-tailed Student’s *t* tests were used. For 3 or more groups, a one-way ANOVA with Dunnett’s post-hoc test (vs. control group) was used. For experiments with multiple groups and longitudinal data, two-way ANOVA, followed by Bonferroni post-hoc test (vs. control for each time point) was performed.

## Results

### Partial dopamine depletion in the bilateral striatal 6-OHDA lesion model

**Fig. 1:**
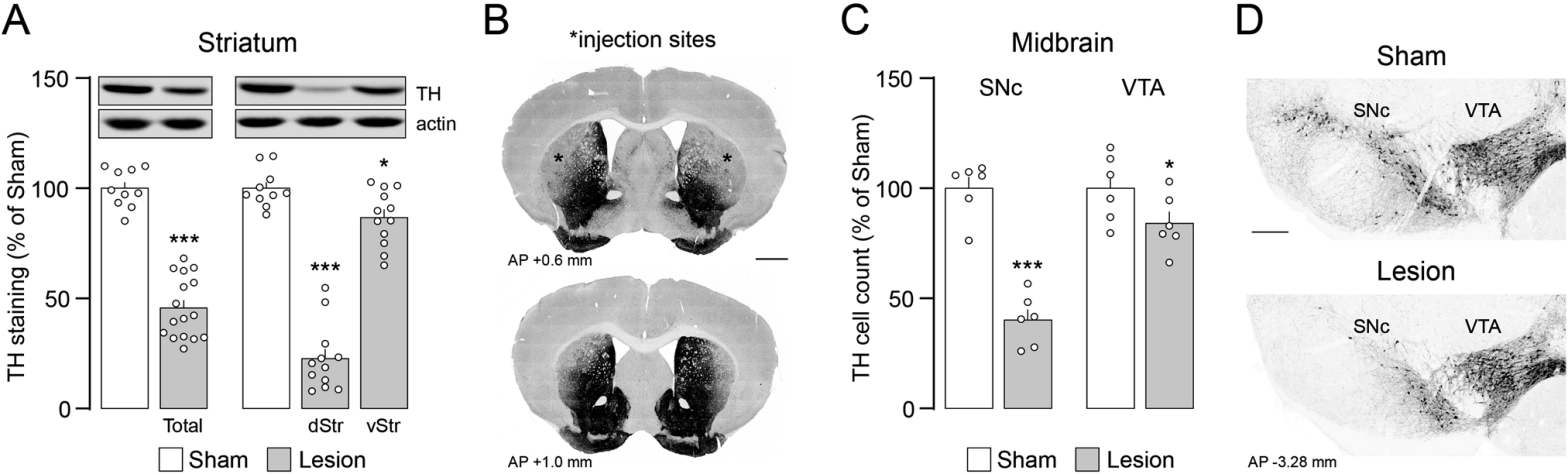
Bilateral partial striatal lesion with 6-OHDA. **A** Western blot showing the percentage of tyrosine hydroxylase (TH)-loss in the striatum of 6-OHDA lesion mice compared to sham mice. Left, total striatum (***p < 0.001 Lesion (n = 16) vs. Sham (n = 10), unpaired *t* test, *t* = 10.96, df = 24). Right, dorsal (dStr) and ventral (vStr) striatum (*p < 0.05 and ***p < 0.001 dStr (n = 12) and vStr (n = 12) vs. Sham (n = 10), one-way ANOVA, Dunnett’s post-hoc test). Upper panels show representative western blots of TH and actin immunoreactivity. **B** Representative brain coronal sections from a 6-OHDA lesion mouse showing immunolabelling of dopaminergic fibers by TH-antibody (black). Upper section, coronal view including the injection site (see stars in the dorso-lateral striatum). Lower section, coronal view rostral to the injection site (AP + 1.0 mm from bregma) including the ventral striatum (nucleus accumbens). Images are presented with inverted color (scale bar 1 mm). **C** Cell count quantifications of TH-positive cells in (left) SNc (n = 6; ***p < 0.001 vs. Sham, unpaired *t* test, *t* = 8.509, df = 10) and (right) VTA (n = 6; *p < 0.05 vs. Sham, unpaired *t* test, *t* = 1.944, df = 10). **D** Representative coronal sections showing TH-positive cells within SNc and VTA of sham and lesion mice (scale bar 250 µm, AP −3.28 mm from bregma). Division of the two areas was based on the axon bundle (medial lemniscus) separating SNc and VTA.

Dopaminergic degeneration after bilateral injection of 6-OHDA in the dorsal striatum was examined 3 weeks post-surgery by western blot and immunohistochemistry using an antibody against TH, the rate-limiting enzyme of catecholamine biosynthesis. Western blot analysis of the whole (total) striatum showed a 54.3% reduction of TH levels in 6-OHDA lesion mice compared to sham lesion mice (Fig. 1A left). 6-OHDA caused a larger reduction of TH levels in the dorsal striatum (77.3%, Fig. 1A right and B upper panel) compared to the ventral striatum (nucleus accumbens) (13.7%, Fig. 1A right and B lower panel). Immunohistochemical analysis of the SNc revealed a 59.8% reduction in the number of dopaminergic cell bodies (Fig. 1C left and D), which mainly project to the dorsal striatum. In the VTA, which preferentially projects to the ventral striatum, dopaminergic cell bodies were less affected (Fig. 1C right and D; 15.9% reduction). In line with previous work [18], we confirmed that the infusion volume and concentration of 6-OHDA (1.25 µl, 4 μg/μl) results in a restricted lesion, limited to the dorsal striatum and its dopaminergic afferents from the SNc.

### 6-OHDA lesion results in L-Dopa-induced place preference and behavioral sensitization

**Fig. 2:**
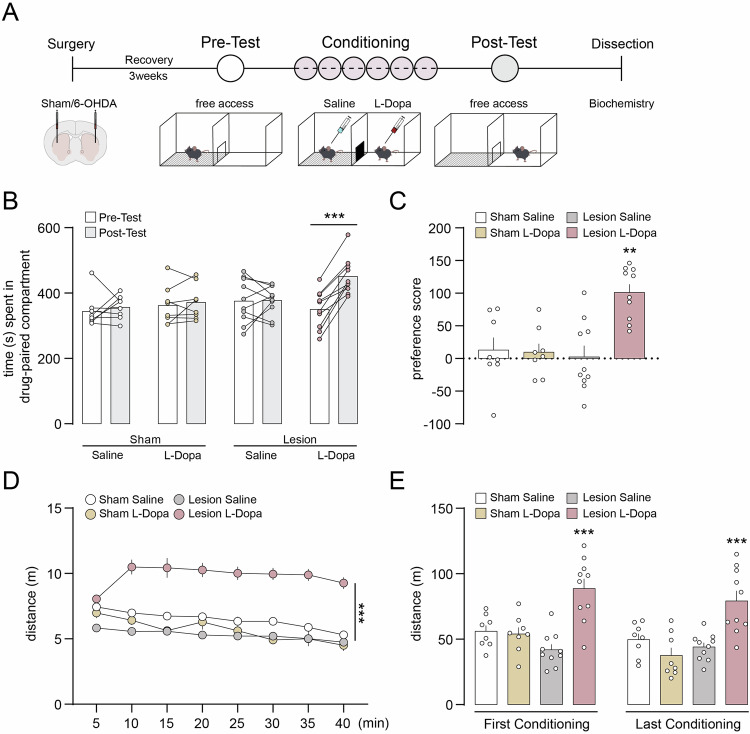
L-Dopa-induced CPP and motor-stimulation in 6-OHDA lesion mice. **A** Schematic illustration of the CPP procedure. Tissue was dissected for biochemistry 24 h after the post-conditioning test. **B** Sham and 6-OHDA lesion mice were treated during the conditioning phase with saline or L-Dopa and CPP was assessed in a drug-free state by comparing the time spent in the drug-paired compartment during pre- and post-conditioning. Sham Saline (n = 8), Sham L-Dopa (n = 8), Lesion Saline (n = 10), Lesion L-Dopa (n = 10). Two-way ANOVA showed a significant effect of time (p < 0.001, *F*_(1, 32)_ = 15.69), no significant effect of group; and significant interaction between group and time (p < 0.001, *F*_(3, 32)_ = 9.423). ***p < 0.001 vs. Pre-Test (Bonferroni post-hoc test). **C** Preference score calculated as post- minus pre-conditioning time spent in the drug-paired compartment. **p < 0.01 vs. Sham Saline (one-way ANOVA, Dunnett’s post-hoc test). **D** Locomotor activity during the conditioning phase was measured as distance (m) moved in drug-paired compartment during the 40 min sessions. Each timepoint shows the average group data of all conditioning sessions. ***p < 0.001 Lesion L-Dopa vs. Sham Saline, Sham L-Dopa, and Lesion Saline (one-way ANOVA with Dunnett’s post-hoc test). **E** Cumulative locomotor activity during the first and last conditioning session. Two-way ANOVA showed a significant effect of groups (p < 0.001, *F*_(3, 32)_ = 16.59), a significant effect of time (p < 0.05, *F*_(1, 32)_ = 6.174, p = 0.0184), but no significant interaction between groups and time. ***p < 0.001 Lesion L-Dopa vs. respective Sham Saline, Sham L-Dopa, or Lesion Saline (Bonferroni post-hoc test).

The rewarding effect of L-Dopa in sham and 6-OHDA lesion mice was examined in the CPP paradigm by measuring the time spent in the saline- or drug-paired compartment during pre- and post-conditioning (Fig. 2A). We found that 6-OHDA mice developed a place preference, as shown by the enhanced time spent in the compartment associated with administration of L-Dopa (Fig. 2B, C). In contrast, sham mice injected with L-Dopa did not display place preference when subjected to the same conditioning protocol. Control groups composed of sham and lesion mice injected with saline in both compartments also did not develop place preference (Fig. 2B, C). In a parallel experiment, we did not observe L-Dopa-induced place preference in 6-OHDA lesion mice 7 days after the last conditioning session (Fig. S1). Altogether, these results indicate that L-Dopa acquires transient rewarding properties only in mice with dopamine depletion.

**Fig. 3:**
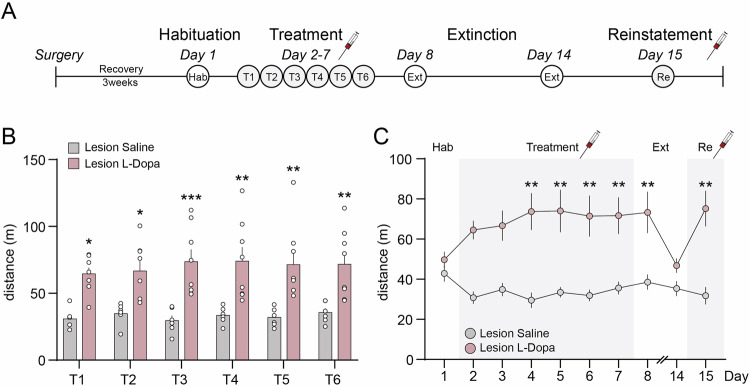
L-Dopa-induced locomotor sensitization in 6-OHDA lesion mice. **A** Schematic illustration of the procedure performed in locomotor activity boxes. **B** Cumulative locomotor activity during the 6 treatment sessions. Two-way ANOVA showed a significant effect of groups (p < 0.01, *F*_(1, 12)_ = 16.40), no significant effect of time and no significant interaction between groups and time. ***p < 0.001, **p < 0.01 and *p < 0.05 Lesion L-Dopa vs. Lesion Saline (Bonferroni post-hoc test). **C** Locomotor activity during Habituation (Hab), Treatment (T), Extinction (Ext) and Reinstatement (Re) phases was measured as distance (m) during 15 min trials. Two-way ANOVA showed a significant effect of groups; Lesion Saline (n = 6) vs. Lesion L-Dopa (n = 8), p < 0.01, *F*_(1, 12)_ = 18.21, no significant effect of time, and significant interaction between groups and time (p < 0.001, *F*_(9, 108)_ = 3.436). **p < 0.01 Lesion L-Dopa Day 4–8 and 15 vs. Day 1 (Bonferroni post-hoc test).

Behavioral sensitization following saline or L-Dopa injection was initially assessed by measuring locomotor activity during the conditioning phase. We found that L-Dopa induced an increase in locomotion only in 6-OHDA lesion mice (Fig. 2D, E). This effect persisted for the entire duration of the 40 min conditioning session (Fig. 2D) and was observed across all conditioning days (Fig. 2E). These results were confirmed by measuring motor activity in locomotor activity boxes (Fig. 3A, B). We found that L-Dopa-induced hyperlocomotion persisted during the 24 h following the last L-Dopa administration. This effect was absent one week after the last treatment but could be reinstated by a single injection of L-Dopa (Fig. 3C).

### L-Dopa-induced signal transduction occurs selectively in the D1R MSN of the dopamine depleted striatum

**Fig. 4:**
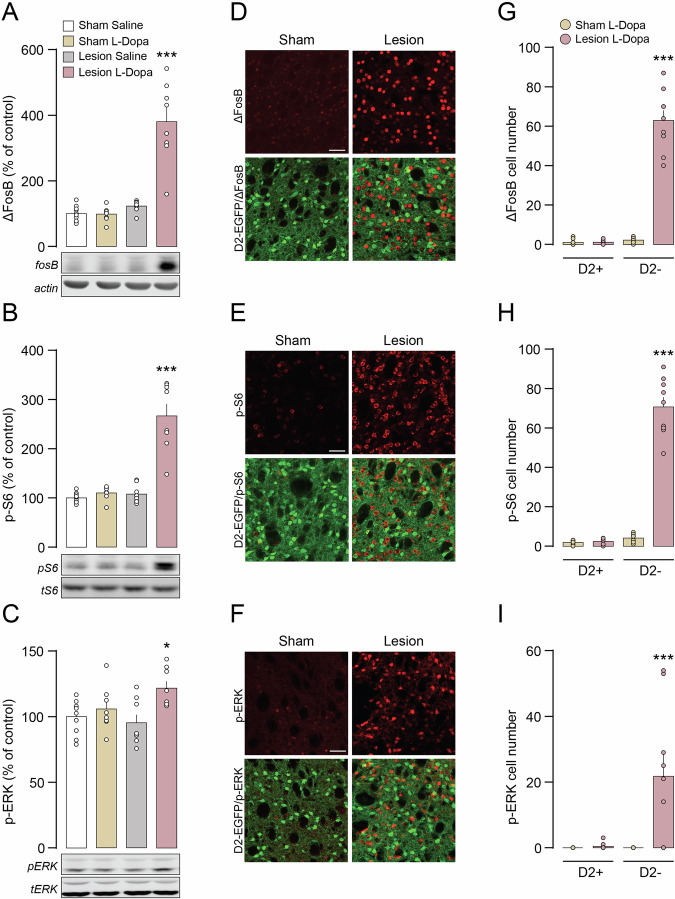
L-Dopa-induced hyperactivation of signaling components occurs selectively in D1R MSN of 6-OHDA lesion mice. Western blot quantification of ΔFosB (**A**), p-S6 (**B**) and p-ERK (**C**) in the striata of Sham and 6-OHDA lesion mice treated with Saline or L-Dopa. Sham Saline (n = 10), Sham L-Dopa (n = 8), Lesion Saline (n = 8), Lesion L-Dopa (n = 8). *p < 0.05 and ***p < 0.001 vs. respective Sham Saline; one-way ANOVA, Dunnett’s post-hoc test. Representative immunostaining of ΔFosB (**D**), p-S6 (**E**) and p-ERK (**F**) (all in red, with EGFP in green) in the dorsal striatum of Sham or 6-OHDA Lesion D2-EGFP mice treated with L-Dopa (scale 50 µm). Quantification of ΔFosB (**G**), p-S6 (**H**) and p-ERK (**I**) positive cells in D2-EGFP positive (D2+) and D2-EGFP negative (D2-) striatal neurons of Sham or 6-OHDA Lesion D2-EGFP mice treated with L-Dopa. Two-way ANOVA (n = 3 animals/group and 3 sections/animal) showed a significant effect of cell type (p < 0.001 *F*_(1, 32)_ = 145.6 for ΔFosB, p < 0.001 *F*_(1, 32)_ = 202.5 for p-S6, and p < 0.01 *F*_(1, 32)_ = 9.184 for p-ERK), group (p < 0.001 *F*_(1, 32)_ = 135.5 for ΔFosB, p < 0.001 *F*_(1, 32)_ = 182.6 for p-S6, and p < 0.01 *F*_(1, 32)_ = 9.966 for p-ERK) and interaction between cell type and group (p < 0.001 *F*_(1, 32)_ = 134.5 for ΔFosB, p < 0.001 *F*_(1, 32)_ = 177.8 for pS6, and p < 0.01 *F*_(1, 32)_ = 9.184 for pERK). ***p < 0.001 vs. respective Sham L-Dopa (Bonferroni post-hoc test).

Next, the effect of L-Dopa on signal transduction within the dorsal striatum was examined. Western blot analysis showed that L-Dopa induced a 4-fold increase of ΔFosB expression in 6-OHDA lesion compared to control mice (Fig. 4A). To study the cellular distribution of ΔFosB in the dorsal striatum, we used transgenic animals expressing enhanced green fluorescent protein (EGFP) under the control of the promoter for the D2R. Quantification of ΔFosB immunolabeling in the 6-OHDA lesion striatum showed nearly exclusive accumulation of ΔFosB in D2-EGFP negative neurons, which correspond to D1R-expressing MSN (Fig. 3D). No increase in ΔFosB expression was observed in control (sham lesion) mice treated with L-Dopa (Fig. 4G). Administration of L-Dopa to 6-OHDA lesion mice also led to a persistent increase in the phosphorylation of the ribosomal protein S6 (p-S6), a downstream effector target of mTORC1 (Fig. 4B) and a similar, albeit less prominent, effect on phosphorylated ERK (p-ERK, Fig. 4C). Immunolabeling of these targets in D2-EGFP mice also confirmed their specific localization in D1R neurons (Fig. 4E, F, H, I). In contrast to the dorsal striatum, administration of L-Dopa did not induce any change in the levels of ΔFosB, or phosphorylated S6 and ERK in the ventral striatum of 6-OHDA lesion mice (Fig. S2). In summary, L-Dopa produced a large accumulation of the transcription factor ΔFosB in 6-OHDA lesion mice, accompanied by hyperactivation of mTORC1 and ERK signaling. This effect was restricted to the dorsal striatum and confined to D1R-expressing neurons.

### L-Dopa-induced CPP and hyperactivation of D1R signaling require intact D1R transmission

**Fig. 5:**
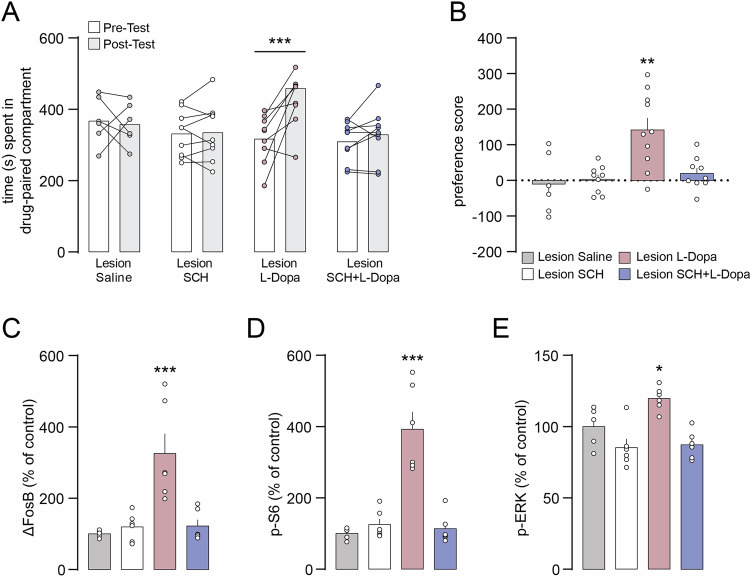
Pharmacological inhibition of D1R prevents L-Dopa-induced CPP and abnormal signal transduction in 6-OHDA lesion mice. **A** 6-OHDA lesion mice were treated during the conditioning phase with saline, SCH23390 (SCH), L-Dopa, or SCH + L-Dopa and CPP was assessed in a drug-free state by comparing the time spent in the drug-paired compartment during pre- and post-conditioning. Lesion Saline (n = 6), Lesion SCH (n = 9), Lesion L-Dopa (n = 10), Lesion SCH + L-Dopa (n = 9). Two-way ANOVA showed a significant effect of time (p < 0.01 *F*_(1, 30)_ = 8.863), no significant effect of group and significant interaction between group and time (p < 0.001, *F*_(3, 30)_ = 8.046). ***p < 0.001 vs. Pre-Test (Bonferroni post-hoc test). **B** Preference score calculated as post- minus pre-conditioning time spent in the drug-paired compartment. **p < 0.01 vs. Lesion Saline (one-way ANOVA, Dunnett’s post-hoc test). Western blot quantification of ΔFosB (**C**), p-S6 (**D**) and p-ERK (**E**) in the striata of 6-OHDA lesion mice treated with saline (n = 5), SCH (n = 6), L-Dopa (n = 6) or SCH + L-Dopa (n = 6). *p < 0.05 and ***p < 0.001 vs. respective Lesion Saline (one-way ANOVA, Dunnett’s post-hoc test).

We next investigated, in 6-OHDA mice, the involvement of D1R in the rewarding effects of L-Dopa and the associated activation of downstream signaling components. Pharmacological inhibition of D1R with the selective receptor antagonist SCH23390 (SCH) abolished the development of CPP (Fig. 5A, B), thereby counteracting the psychostimulant-like effect of L-Dopa. Importantly, SCH also prevented the accumulation of ΔFosB and the concomitant hyperactivation of mTORC1 and ERK signaling (Fig. 5C–E). Administration of SCH in the absence of L-Dopa did not induce place preference or biochemical changes. Thus, in parkinsonian mice, pharmacological inhibition of D1R prevents L-Dopa-induced place preference and associated abnormal signaling.

### Chemogenetic inhibition of D1R prevents the development of CPP

**Fig. 6:**
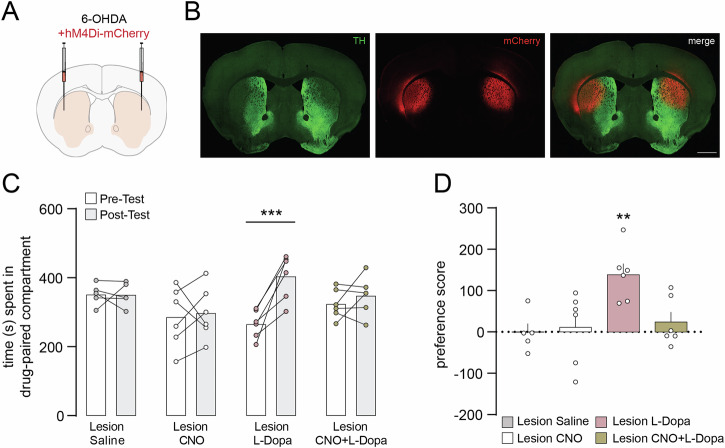
Chemogenetic inhibition of D1R-expressing neurons in 6-OHDA lesion mice prevents L-Dopa-induced CPP. **A** Injection site of the AAV5 vector used to express Gi-DREADD in the dorsal striatum of D1-Cre mice with a bilateral 6-OHDA lesion. **B** Representative immunofluorescence image showing the distribution of TH-immunoreactivity (green) and mCherry-positive cells (red) three weeks after AAV injection in the dorso-lateral striatum of the 6-OHDA lesion mice. Scale bar 1 mm. **C** 6-OHDA lesion mice expressing Gi-DREADD in the dorsal striatum were treated during the conditioning phase with saline, CNO, L-Dopa, or CNO + L-Dopa and CPP was assessed in a drug-free state by comparing the time spent in the drug-paired compartment during pre- and post-conditioning. Lesion Saline (n = 5), Lesion CNO (n = 6), Lesion L-Dopa (n = 6), Lesion CNO + L-Dopa (n = 6). Two-way ANOVA showed a significant effect of time (p < 0.01, *F*_(1, 19)_ = 9.491), no significant effect of group and significant interaction between group and time (p < 0.01, *F*_(3, 19)_ = 5.370). ***p < 0.001 vs. Pre-Test (Bonferroni post-hoc test). **D** Preference score calculated as post- minus pre-conditioning time spent in the drug-paired compartment. **p < 0.01 vs. Lesion Saline (one-way ANOVA, Dunnett’s post-hoc test).

To confirm the involvement of abnormal striatal transmission in L-Dopa-induced place preference, we selectively suppressed the activity of D1R-expressing MSN with inhibitory DREADD. To validate this approach, we first verified that CNO-mediated inhibition of D1R MSN expressing hM4Di in the dorsal striatum suppressed L-Dopa-induced contralateral rotations in unilateral lesion D1-Cre mice (Fig. S3). Immunolabelling against TH and mCherry (tagged to DREADD) showed that DREADD expression was localized in the dopamine-depleted dorsal striatum (Fig. 6A, B and Fig. S3). In line with the results obtained with SCH23390, the selective chemogenetic inhibition of D1R MSN prevented L-Dopa-induced place preference in bilateral 6-OHDA lesion mice (Fig. 6C, D).

### Pharmacological inhibition of mTORC1 and ERK does not prevent the development of CPP

**Fig. 7:**
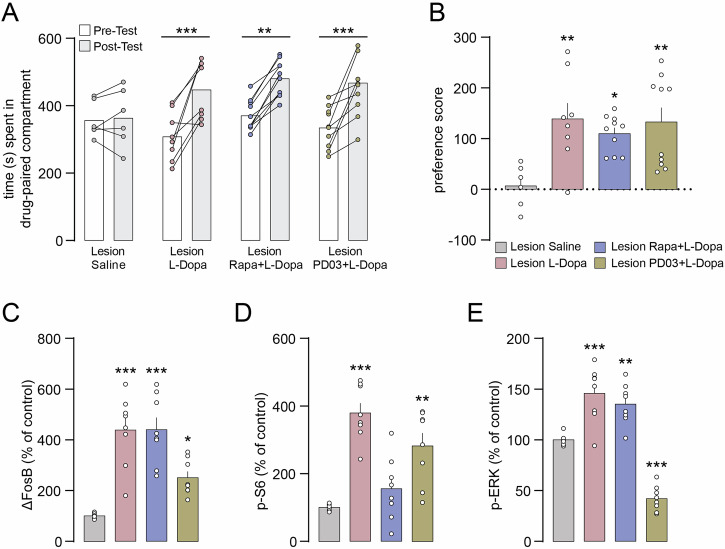
Inhibition of mTORC1 or ERK does not prevent L-Dopa-induced CPP or the accumulation of ΔFosB in the dorsal striatum of 6-OHDA lesion mice. **A** 6-OHDA lesion mice were treated during the conditioning phase with saline, L-Dopa, Rapamycin (Rapa) + L-Dopa, or PD0325901 (PD03) + L-Dopa and CPP was assessed in a drug-free state by comparing the time spent in the drug-paired compartment during pre- and post-conditioning. Lesion Saline (n = 6), Lesion L-Dopa (n = 8), Lesion Rapa + L-Dopa (n = 10), Lesion PD03 + L-Dopa (n = 10). Two-way ANOVA showed a significant effect of time (p < 0.001, *F*_(1, 30)_ = 50.40), no significant effect of group and significant interaction between group and time (p < 0.01, *F*_(3, 30)_ = 5.793). **p < 0.01 and ***p < 0.001 vs. respective Pre-Test (Bonferroni post-hoc test). **B** Preference score calculated as post- minus pre-conditioning time spent in the drug-paired compartment. *p < 0.05 and **p < 0.01 vs. Lesion Saline (one-way ANOVA, Dunnett’s post-hoc test). Western blot quantification of ΔFosB (**C**), p-S6 (**D**) and p-ERK (**E**) in the striata of 6-OHDA lesion mice treated with saline (n = 6), L-Dopa (n = 8), Rapa + L-Dopa (n = 8), or PD03 + L-Dopa (n = 8). *p < 0.05, **p < 0.01 and ***p < 0.001 vs. respective Lesion Saline (one-way ANOVA, Dunnett’s post-hoc test).

We next examined whether the abnormal activation of mTORC1 and ERK was implicated in the rewarding properties of L-Dopa in our mouse model of PD. Inhibition of mTORC1 with rapamycin, or ERK with PD0325901 during L-Dopa-conditioning did not prevent the development of CPP (Fig. 7A, B). Similarly, we did not observe any change in L-Dopa-induced place preference when rapamycin and PD0325901 were administered before the post-conditioning test to prevent memory recall (Fig. S4).

Western blot analysis showed that administration of rapamycin was able to block the L-Dopa-mediated increase in phosphorylated S6 (Fig. 7D), but had no effect on the accumulation of ΔFosB and only a minor effect on phosphorylated ERK levels (Fig. 7C, E). The selective ERK antagonist PD0325901 reduced phospho-ERK levels below baseline (Fig. 7E), but only partially reduced the accumulation of ΔFosB in 6-OHDA lesion mice (Fig. 7C). These results indicate that inhibition of abnormally activated upstream signaling targets is not sufficient to suppress the rewarding effect of L-Dopa in PD and point to persistent accumulation of ΔFosB as a possible culprit.

## Discussion

The main finding of this study is that, in a toxin-based mouse model of PD, L-Dopa acquires rewarding properties reminiscent of DDS, which are causally linked to the activation of sensitized D1R in the dorsal striatum. In the same model, we also show that administration of L-Dopa results in abnormal striatal signaling leading to increased levels of ΔFosB, which is counteracted by pharmacological inhibition of D1R.

We show that, in contrast to control mice, 6-OHDA lesion mice tested in the CPP behavioral paradigm develop a preference for the environment associated with the administration of L-Dopa. These results are congruent with clinical reports of PD patients experiencing feelings of pleasure and well-being in response to L-Dopa [24]. They are also in line with the observation that administration of L-Dopa to healthy subjects is not associated with pleasurable or mood elevating effects [25], thereby indicating that a pre-existent loss of striatal dopamine innervation is necessary for L-Dopa to acquire psychostimulant-like properties.

The ability of L-Dopa to induce CPP in our bilateral 6-OHDA lesion mice is in agreement with previous behavioral results obtained in a PD rat model generated by overexpression of α-synuclein in the SNc [16]. However, in other rat models based on 6-OHDA lesion of the striatum or the medial forebrain bundle, L-Dopa failed to show rewarding effects [26, 27]. Aside from possible species-specific differences, this discrepancy may be explained by the distinct CPP protocols employed. In particular, the number of conditioning sessions and their duration following drug or vehicle administration were increased in comparison to the above-mentioned studies. This procedure was used to decrease the interference of reduced memory function observed in the same mouse model [28] and reported also in other rodent models of PD [29]. Memory impairment may also be responsible for the lack of L-Dopa-induced place preference observed after a 7-day interruption of administration. In addition, the more transient profile of CPP in the PD model may be related to the limited action of L-Dopa in the ventral striatum, which is instead affected by conventional addictive drugs. The inability of L-Dopa to affect dopamine signaling components in this region depends on the mild impact of 6-OHDA on the mesolimbic pathway, which recapitulates the clinical condition, and results in insufficient sensitization of dopamine receptors.

The dopamine D2/D3 receptor agonist, pramipexole, displayed reinforcement effects in several rat models of PD. However, differently from L-Dopa, the rewarding properties of D2/D3 receptor agonists were also observed in naïve rats and were further increased following lesion of the nigrostriatal system [27, 30, 31]. This exacerbation was paralleled by augmented expression of ΔFosB in the dorsal striatum, occurring in both D1 and D2 receptors [31], which contrasts with the more selective effect on ΔFosB, limited to D1R-expressing neurons, produced by L-Dopa in the 6-OHDA mouse model. Taken together, these findings indicate that the ability of L-Dopa to gain psychostimulant-like properties differs from that of pramipexole by being more strictly dependent on the loss of dopamine in the dorsal striatum, leading to sensitized D1R-mediated transmission.

Our results indicate that the ability of L-Dopa to induce place preference in 6-OHDA lesion mice is prevented by co-administration of the D1R antagonist, SCH23390. Importantly, the rewarding profile of L-Dopa is also abolished by DREADD-mediated silencing of D1R MSN within the dorsal striatum. Taken together, these findings provide the first evidence of a causal link between abnormal activation of D1R-expressing MSN and L-Dopa-induced addictive-like behavior.

The involvement of D1R in the rewarding properties of dopamine replacing agents has been challenged by a study showing that administration of the D1R agonist, SKF81297, induces place preference in naïve rats, but exerts instead an aversive effect in rats with a combined 6-OHDA lesion of the SNc and VTA [27]. This opposite response may depend on the abnormal stimulation produced by SKF81297 acting on the sensitized D1R of 6-OHDA lesion rats. Indeed, the rewarding effect of SKF81297 measured in the CPP test is biphasic, with large doses failing to produce a significant place preference [32]. Along these lines of thought, the rewarding properties displayed in 6-OHDA lesion mice by L-Dopa are likely dependent on its ability to activate D1R within a range similar to that achieved in normal mice by a moderate dose of D1R agonists.

The D1R-dependent increase of striatal ΔFosB expression produced by L-Dopa in the PD mouse model is accompanied by activation of the ERK and mTORC1 signaling pathways. Inhibitors of these intracellular cascades have been shown to counteract motor complications induced by L-Dopa, i.e., dyskinesia [33, 34], but here they did not prevent L-Dopa-induced place preference, independently of whether administered during conditioning, or as a single injection before the post-conditioning test, to examine a possible effect on memory recall. Interestingly, administration of L-Dopa in combination with ERK or mTORC1 inhibitors failed to normalize ΔFosB levels. Considering the well-established role ΔFosB in drug addiction [14] it is possible that the rewarding properties displayed by L-Dopa in 6-OHDA lesion mice are linked to persisting abnormal expression of this transcription factor.

The present results show that, in 6-OHDA lesion mice, the increased responsiveness of D1R to administration of L-Dopa is accompanied by a motor-stimulant effect. In naïve animals, this behavioral sensitization is regarded as a marker of drug-seeking and is generated gradually by repeated, intermittent administration of substances of abuse [35]. Notably, in the 6-OHDA PD model, the enhanced motor-stimulant response was already observed after the first administration of L-Dopa and persisted unchanged during the entire course of the CPP test. In line with previous work on addictive drugs [35–37], we found that context-dependent motor-activation persisted for up to 24 h after L-Dopa administration and could be reinstated by a single administration following extinction. Moreover, the rapid onset of behavioral sensitization is an important correlate to the sensitization of D1R observed in 6-OHDA lesion mice and represents an additional factor conferring rewarding properties to L-Dopa.

The PD model utilized in this study is characterized by a prevalent loss of SNc neurons projecting to the dorsal striatum with only a minor reduction in the number of VTA neurons projecting to the nucleus accumbens [18]. This neurodegenerative pattern is in line with that reported in PD patients [38, 39] and is consistent with the sensitization of D1R and the consequent hyperactivation of dopamine transmission produced by L-Dopa in the dorsal striatum. The implication of these abnormalities in the rewarding properties exhibited by L-Dopa in the PD model suggests that therapeutic approaches targeting excessive D1R-mediated transmission may be beneficial for the treatment of DDS.

## Supplementary information

Supplementary Material

## Data Availability

Data from this study are available from the authors upon reasonable request.
