# Supplementary material for: Rewarding properties of L-Dopa in experimental parkinsonism are mediated by sensitized dopamine D1 receptors in the dorsal striatum
Source: Mol Psychiatry. 2024 Sep 3;30(3):976–85. doi: 10.1038/s41380-024-02721-3 (PMC11835726; doi:10.1038/s41380-024-02721-3)
Supplement: Supplementary file 1 — Supplementary Material [file 41380_2024_2721_MOESM1_ESM.pdf]

Supplementary Information

Rewarding properties of L-Dopa in experimental parkinsonism are mediated by sensitized dopamine D1 receptors in the dorsal striatum

Carina Plewnia[1] Ph.D., Débora Masini[1,2] Ph.D., Gilberto Fisone[1] Ph.D.

[1] *Department of Neuroscience, Karolinska Institutet, 17165 Stockholm, Sweden*

[2] *Current Affiliation: Department of Biochemistry and Biophysics, Stockholm University, 10691 Stockholm, Sweden*

Corresponding author: Gilberto Fisone, Department of Neuroscience, Karolinska Institutet, Solnavägen 9, 17165 Solna, Sweden, Tel. +46852487375, gilberto.fisone@ki.se

Supplementary Figures S1-S4

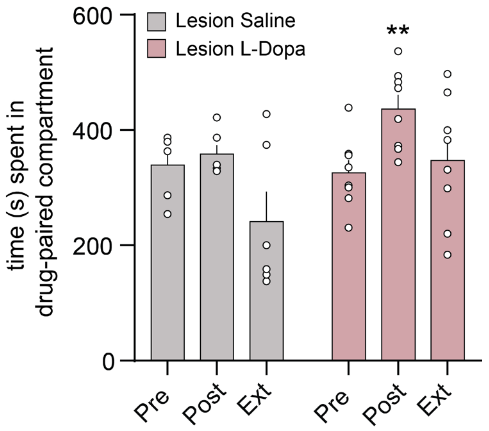

Figure S1. Lack of L-Dopa-induced CPP at 7 days after conditioning. 6-OHDA lesion mice were treated during the conditioning phase with saline or L-Dopa and CPP was assessed in a drug-free state by comparing the time spent in the drug-paired compartment during pre-conditioning (Pre) and post-conditioning at 24 h (Post) and 7 days (Ext). Lesion Saline (n = 6), Lesion L-Dopa (n = 8). Two-way ANOVA showed a significant effect of time ($p < 0.01$, $F_{(1.365, 16.37)} = 8.399$), no significant effect of group and no significant interaction between group and time. **$p < 0.01$ vs. Pre-Test (Bonferroni post-hoc test).

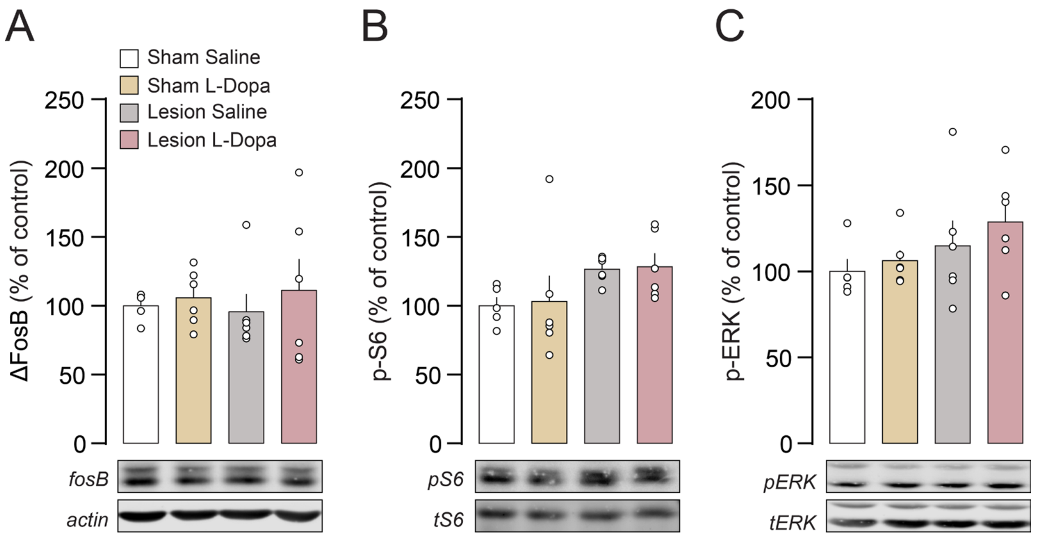

Figure S2. Lack of L-Dopa effect on signal transduction within the ventral striatum. Western blot quantification of ΔFosB (A), p-S6 (B) and p-ERK (C) in the ventral striata of Sham and 6-OHDA lesion mice treated with saline or L-Dopa. Sham Saline (n = 5), Sham L-Dopa (n = 6), Lesion Saline (n = 6), Lesion L-Dopa (n = 6). One-way ANOVA with Dunnett's post-hoc test vs. respective Sham Saline.

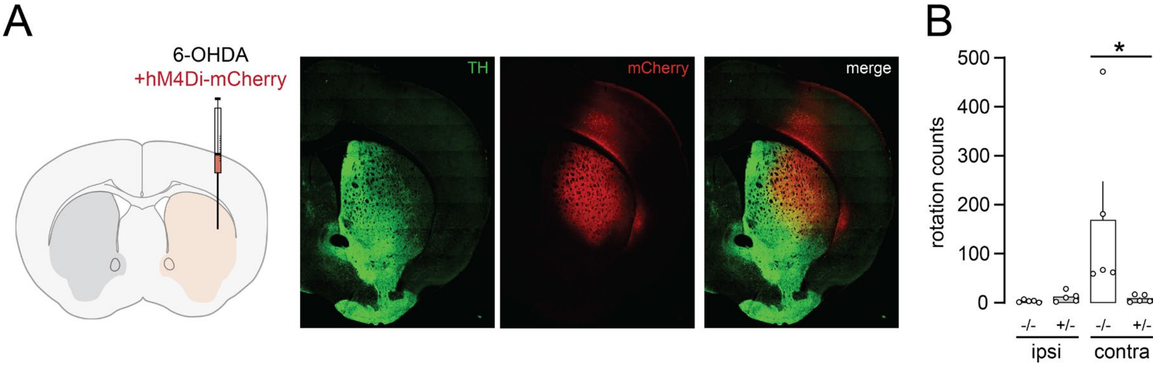

Figure S3. Chemogenetic inhibition of D1R-expressing neurons in unilateral 6-OHDA lesion mice prevents L-Dopa-induced contralateral rotation. (A) Left, injection site of the AAV5 vector used to express Gi-DREADD in the dorsal striatum of D1-Cre mice with a unilateral 6-OHDA lesion. Right, representative immunofluorescence image showing the distribution of TH- (green) and mCherry- (red) positive cells three weeks after injection of AAV in the dorso-lateral striatum of the 6-OHDA lesion hemisphere. (B) Cre-negative (-/-) control, and Cre-positive (+/-) Gi-DREADD-expressing mice were treated with L-Dopa + CNO and ipsilateral (ipsi) and contralateral (contra) rotations were counted. *p > 0.05 vs. contralateral rotations in Cre +/- mice (n = 5, unpaired t-test, t = 2.015, df = 8).

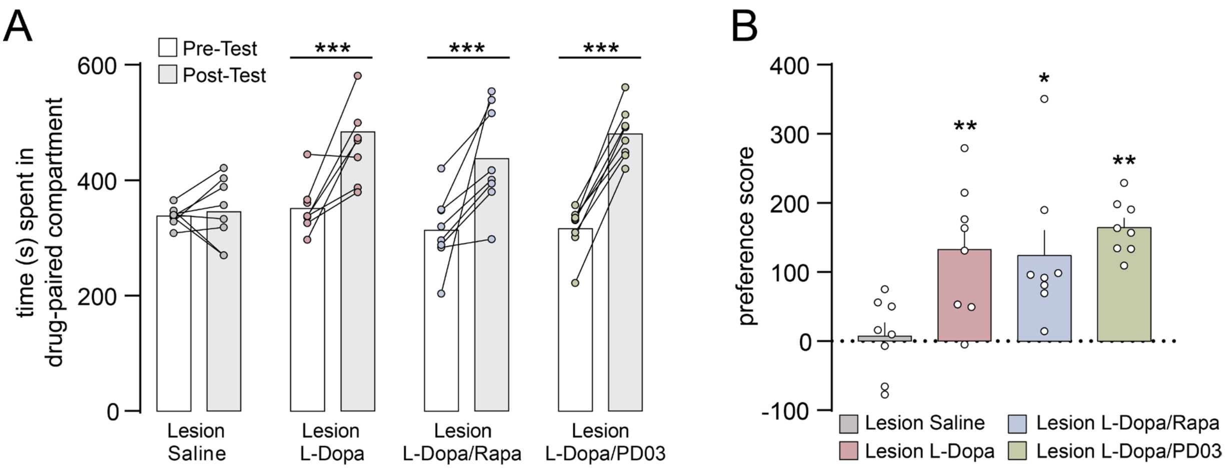

Figure S4. Inhibition of mTORC1 or ERK does not prevent CPP memory recall. (A) 6-OHDA lesion mice were conditioned with saline or L-Dopa and treated with vehicle, rapamycin (Rapa), or PD0325901 (PD03) 45 min before the post-conditioning test and CPP was assessed by comparing the time spent in the drug-paired compartment during pre- and post-conditioning. All groups n = 8. Two-way ANOVA showed a significant effect of time ($p < 0.001$, $F_{(1, 28)} = 60.18$), a significant effect of group ($p < 0.05$, $F_{(1, 28)} = 3.928$) and significant interaction between group and time ($p < 0.01$, $F_{(3, 28)} = 6.221$). ***p < 0.001 vs. respective Pre-Test (Bonferroni post-hoc test). (B) Preference score calculated as post- minus pre-conditioning time spent in the drug-paired compartment. *p < 0.05 and **p < 0.01 vs. Lesion Saline (one-way ANOVA, Dunnett's post-hoc test).
